# The impact of functional *MDM2*-polymorphisms on neutrophil counts in breast cancer patients during neoadjuvant chemotherapy

**DOI:** 10.1186/s12885-025-13675-2

**Published:** 2025-02-20

**Authors:** Nora D. Hatletvedt, Christina Engebrethsen, Jürgen Geisler, Stephanie Geisler, Turid Aas, Per E. Lønning, Liv B. Gansmo, Stian Knappskog

**Affiliations:** 1 Department of Clinical Science, University of Bergen, 5020 Bergen, Norway; 2 Department of Oncology, Haukeland University Hospital, Bergen, Norway; 3 Department of Oncology, Akershus University Hospital, Lørenskog, Norway; 4 Institute of Clinical Medicine, University of Oslo, Oslo, Norway; 5 Department of Surgery, Haukeland University Hospital, Bergen, Norway

**Keywords:** MDM2 polymorphism, Breast cancer, Neutropenia, Chemotherapy

## Abstract

**Background:**

Functional polymorphisms in the *MDM2* promoters have been linked to cancer risk and several non-malignant conditions. Their potential role in bone marrow function during chemotherapy is largely unknown.

**Methods:**

We investigated the potential associations between genotypes of *MDM2* SNP309 (rs2279744), SNP285 (rs117039649) and del1518 (rs3730485) and neutrophil counts in breast cancer patients receiving neoadjuvant sequential epirubicin and docetaxel, with additional G-CSF, in the DDP-trial (NCT00496795). We applied longitudinal ratios, post vs. pre-treatment, of neutrophil counts as our main measure. Differences by genotypes were tested by Jonckheere-Terpstra test for ranked alternatives, while dominant and recessive models were tested by Mann–Whitney U test, and additional sub-analyses were performed for genotype combinations.

**Results:**

The SNP309 reference T-allele was associated with a better sustained neutrophil count (*p* = 0.035). A similar association was observed for the alternative del-allele of the del1518 (*p* = 0.049). Additionally, in combined genotype-analyses, patients with the SNP309 TT genotype and at least one copy of the del1518 del-allele had particularly favorable sustained neutrophil counts during chemotherapy treatment (*p* = 0.005).

**Conclusions:**

Our study provides evidence that *MDM2* promoter polymorphisms may be associated with neutrophil counts and bone marrow recovery during chemotherapy treatment in breast cancer patients.

**Trial registration:**

The DDP-trial was registered at ClinicalTrials.gov (NCT00496795; registration date 2007–07-04).

**Supplementary Information:**

The online version contains supplementary material available at 10.1186/s12885-025-13675-2.

## Background

Murine Double Minute 2 (MDM2) is an ubiquitin ligase regulating p53 activity in an auto-regulatory feedback loop [1–5]. Under normal conditions, p53 levels are kept low due to ubiquitination by MDM2, while in stressed cells, p53 accumulates and transactivates downstream target genes that, in turn, may induce cell cycle arrest, DNA damage repair, senescence, or apoptosis [6]. MDM2 may also interact with proteins involved in cell cycle regulation independently of *p53*, as shown for p21, FOXO3a, XIAP and Nbs1 [7–10].

The *MDM2* gene has two separate promoters (P1 and P2), initiating expression of transcripts with distinct first exons, both sharing the same translation start site [11, 12]. Several functional polymorphisms have been identified in the *MDM2* promoters. Thus, the *MDM2* SNP309 (rs2279744) G-allele results in an elongated binding site for the transcription factor Sp1, leading to increased *MDM2* expression, and several studies have linked the G-allele to an increased risk of various cancer forms [13–15]. In contrast, the variant C-allele of the *MDM2* SNP285 (rs117039649), occurring in complete linkage disequilibrium with 309G, reduces the binding affinity of Sp1 to another binding site within the same promoter and has been found associated with a reduced risk of breast, ovarian and endometrial cancer [16, 17]. Finally, the del1518 variant (rs3730485) is a 40 bp deletion that removes a putative TATA motif [18, 19] and this variant has been linked to an increased risk of hepatocellular and colon cancer, as well as a decreased risk of endometrial cancer, among individuals carrying the SNP309TT genotype [20–22].

Some studies have assessed the role of polymorphisms in the *MDM2-TP53* pathway in contexts beyond that of malignant disease. Gansmo et al. found the *MDM2* SNP309 G-allele to be associated with a reduced risk of rheumatoid arthritis while the *MDM2* del1518 del-allele was associated with an increased risk [23]. Further, findings by Jacovas and colleagues suggest the observed differences in the distribution of the *MDM2* SNP309 genotypes between populations to be a result of diverse selection pressures, with the SNP309 TT genotype being selected for among people living at high altitudes [24]. However, this has partly been disputed in a study on the distribution of *MDM2* SNP309 and *TP53* Arg72Pro in relation to various climate variables [25]. Regarding bone marrow function, *MDM2* SNP309 and *TP53* Arg72Pro have been linked to febrile neutropenia in breast cancer patients receiving a combinatorial 5FU/epirubicin/cyclophosphamide (FEC) regimen, with those patients harboring the SNP309GG genotype having a lower incidence of severe neutropenia when receiving treatment in the highest dose range [26]. Notably, all the studies above that have assessed the relationship between *MDM2* and bone marrow function, have only considered the *MDM2* SNP309 polymorphism and not investigated the effect of *MDM2* SNP285 and *MDM2* del1518, or the combinatorial effects of several polymorphisms in haplotypes.

Bone marrow toxicity is a frequent side effect limiting optimal use of chemotherapy in cancer patients, sometimes causing life-threatening co-morbidity [27]. In this study, we investigate the *MDM2* polymorphisms SNP309, SNP285 and del1518 for potential impact on hematopoietic recovery, focusing on neutrophil counts during neoadjuvant chemotherapy treatment with sequential monotherapy of epirubicin and docetaxel in patients with primary breast cancer.

## Methods

### Patients and samples

**Fig. 1:**
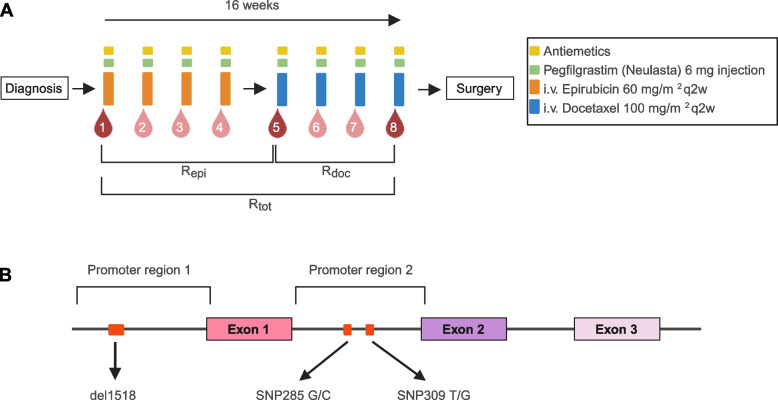
**A** Chemotherapy schedule and blood sampling. Patients with locally advanced breast cancer received neoadjuvant chemotherapy with four cycles of i.v. epirubicin 60 mg/m^2^ q2w (orange boxes) followed by four cycles of docetaxel 100 mg/m^2^ q2w (blue boxes). Blood samples (red drops) for standard clinical biochemical analyses and differential leukocyte count were drawn prior to each of the eight chemotherapy cycles. Bone marrow function was assessed based on neutrophil cell counts at chosen timepoints during treatment. Ratio during epirubicin treatment was calculated as ratio of values from blood sample 5 divided by values from blood sample 1 (R_epi_). Similarly, the ratio for treatment with docetaxel (R_doc_) was defined as sample 8/sample 5 while the ratio for the complete treatment (R_tot_) was defined as sample 8/sample 1. In parallel, patients also received G-CSF (pegfilgrastim) and antiemetics according to hospital guidelines. Illustration created with BioRender.com. **B** *MDM2* promoter regions. Schematic overview of the *MDM2* promoter regions, with the functional *MDM2* polymorphisms indicated in red. *MDM2* del1518 is a 40 bp deletion located in promoter P1, and *MDM2* SNP285 and *MDM2* SNP309 are located in promoter P2. Illustration created with BioRender.com.

All samples in the present study were drawn from patients treated for large primary breast cancers as part of a phase II investigator-initiated single arm study applying dose-dense (2-weekly) treatment with 4 cycles of epirubicin (60 mg/m^2^) followed by 4 cycles of docetaxel (100 mg/m^2^; Fig. 1A) conducted at Haukeland University Hospital between 2007 and 2016 (Dose Dense Protocol [DDP]; NCT00496795, registration date 2007–07-03, ClinicalTrials.gov). The trial details have been described previously [28]. In brief, the trial enrolled 109 patients with non-inflammatory, primary breast cancers with a tumor size > 4 cm and/or N2-3 regional lymph node metastases. The primary goal was to identify factors predicting response to dose-intensive sequential neoadjuvant epirubicin and docetaxel therapy based on genomic analyses of sequential tumor tissue samples.

All patients received bone marrow support, G-CSF (Neulasta®; pegfilgrastim) as a 6 mg subcutaneous injection 24–48 h after each chemotherapy cycle. Patients were treated with antiemetics according to hospital guidelines: During the epirubicin treatment this consisted of ondansetron, dexamethasone and aprepitant, while during the docetaxel treatment a combination of ondansetron and dexamethasone was used. Blood samples for standard clinical biochemical analyses and differential leukocyte count were drawn prior to each of the eight chemotherapy cycles.

Among 109 patients in the trial, 1 patient was excluded from the present analyses since she only completed three courses of epirubicin and had no docetaxel treatment. Out of the 108 patients included for analyses, 92 completed the entire neoadjuvant treatment as per protocol. For 13 patients, docetaxel treatment was not completed due to side effects, and 12 of these patients were only included in the calculations related to epirubicin, while the remaining 1 patient could be included in all analyses since a blood sample was drawn prior to the planned eighth cycle, even if the eighth cycle was not completed. The remaining 3 patients either progressed on epirubicin treatment (*n* = 2) or received alternative chemotherapy in the first four courses on suspicion of metastases (*n* = 1). These 3 were only included in the present study for calculations related to docetaxel. For details, see Supplementary Fig. 1 and Supplementary Table 1.

### Blood count and measures of bone marrow function

Blood samples were drawn and analyzed for hematological parameters before each of the eight chemotherapy cycles. We assessed neutrophil cell counts as our main measure. Secondary measures were thrombocyte counts and hemoglobin values. Further, we performed analyses using total leukocyte cell counts (which should in theory be dominated by neutrophils after G-CSF treatment). We defined the ratio of change in cell count during epirubicin treatment (R_epi_) as cell count in blood samples before chemotherapy cycle 5 (first cycle of docetaxel) divided by the corresponding count before cycle 1 (baseline value, prior to any chemotherapy; Fig. 1A). The ratio of change in cell count during docetaxel treatment (R_doc_) was defined as cell count in blood samples before cycle 8 of chemotherapy divided by the corresponding count before cycle 5. The ratio of change over the total neoadjuvant treatment (R_tot_) was defined as cell count before cycle 8 divided by the corresponding count before cycle 1.

### Genotyping of *MDM2* polymorphisms

All 108 patients were genotyped for three functional polymorphisms in *MDM2*: SNP309 T > G (rs2279744; GRCh38: chr12:68,808,800, SNP285 G > C (rs117039649; chr12:68,808,776) and del1518 ins/del40 (rs3730485; chr12:68,807,065). For a schematic overview, see Fig. 1B. Genotyping of *MDM2* SNPs 285 and 309 was performed by LightSNiP technology (TIB-Molbiol; Berlin, Germany), as previously described [29]. In brief, 10–50 ng DNA was added to a reaction mix of 1 μl LightCycler FastStart DNA Master HybProbe mix (Roche; Basel, Switzerland), 0.5 μl LightSNiP mix (TIB-Molbiol) and 3 mM MgCl_2_, in a final volume of 10 μl. Thermocycling and melt curve conditions were identical for both LightSNiP assays and set according to the optimized protocol provided by TIB-Molbiol. Thermocycling conditions were: 10 min of initial denaturation at 95 °C, 45 cycles of denaturation at 95 °C for 10 s, annealing at 60 °C for 10 s, and elongation at 72 °C for 15 s. A melting curve profile for each sample was obtained by high-resolution melting from 40 °C to 75 °C with a ramp rate of 1.5 °C per s, then cooling at 40 °C for 30 s. Melt Curve Genotyping was performed using the LightCycler 480 software. For quality control, 5% of samples were genotyped by sanger sequencing as previously described elsewhere [16].

Del1518 was genotyped as previously described [22]. In brief, the region of the *MDM2* promoter P1 containing the del1518 was amplified by PCR using the VWR Taq DNA Polymerase system (VWR; Radnor, Pennsylvania, USA), with the primer pair described by Dong et al., 2012 [21]. The amplification was performed in a total reaction volume of 25 μl, containing 2.5 μl 10 × Key Buffer, 0.5 mM dNTPs, 0.5 μM of each primer, 0.25 μl Taq polymerase and 1 μl template DNA (10–100 μg). The thermocycling conditions were an initial step of 94 °C for 5 min, 35 cycles of 94 °C for 30 s, 58 °C for 30 s and 72 °C for 30 s, followed by a final elongation step of 72 °C for 10 min. The PCR products were separated by electrophoresis in a 3% agarose gel and visualized by GelRed Nucleic Acid Gel Stain (BIOTIUM; Fremont, California, USA). The del1518 insertion and deletion alleles were observed as 481 base pairs and 441 base pair bands, respectively.

### Statistical analysis

Potential differences in calculated ratios of neutrophil counts and secondary measures (R_tot_, R_epi_, R_doc_) with respect to genotypes (3 groups) were tested by Jonckheere-Terpstra test for ranked alternatives, while dominant and recessive models (2 groups) were tested by Mann–Whitney U test. Notably, due to the relatively close proximity of the *MDM2* polymorphisms (Fig. 1B), they are in strong linkage disequilibrium to each other. Therefore, sub-analyses were performed for genotype combinations (e.g., since the SNP285 C-allele only occurs on the SNP309 G-allele, analyses for SNP285 C were performed within the subgroup of patients with the SNP309 GG and/or TG genotypes etc.). All statistical analyses were performed using the IBM SPSS statistics (version 27/28) software package for windows. All p-values are given as two-sided and *p* < 0.05 was considered significant.

## Results

### *MDM2 *promoter polymorphism genotypes

Genotyping blood DNA from 108 breast cancer patients, we found the minor allele frequencies (MAF) for *MDM2* SNP309 (rs2279744), SNP285 (rs117039649) and del1518 (rs3730485) to be 0.36, 0.05 and 0.37, respectively. The genotype distributions were as expected based on previous findings [30], and all three polymorphisms were in Hardy–Weinberg equilibrium (Supplementary Table 2). Further, the intraindividual distributions of genotypes were as expected from previous findings of linkage disequilibrium [22, 31]. I.e., SNP285 C-alleles were only detected in patients with at least one SNP309 G-allele and del1518 del-alleles were only detected in patients with at least one SNP309 T-allele (Supplementary Table 3).

The genotype groups were equally balanced for age and body mass index (BMI) (Supplementary Table 4). Importantly, no significant correlations were observed between age or BMI, two possible factors influencing bone marrow function, and pretreatment neutrophil measures used in the present analyses (Supplementary Table 5).

### *MDM2* SNP309 status and neutrophil count during chemotherapy

To assess the impact of *MDM2* promoter polymorphisms on neutrophil counts during neoadjuvant chemotherapy, we applied ratios of neutrophil count after treatment with both drugs vs. before treatment start (R_tot_), after vs. before epirubicin (R_epi_), and after vs. before docetaxel (R_doc_; for overview see Methods and Fig. 1A. For neutrophil counts and calculated ratios, see Supplementary Table 6 and Supplementary Fig. 2A and B).

**Fig. 2:**
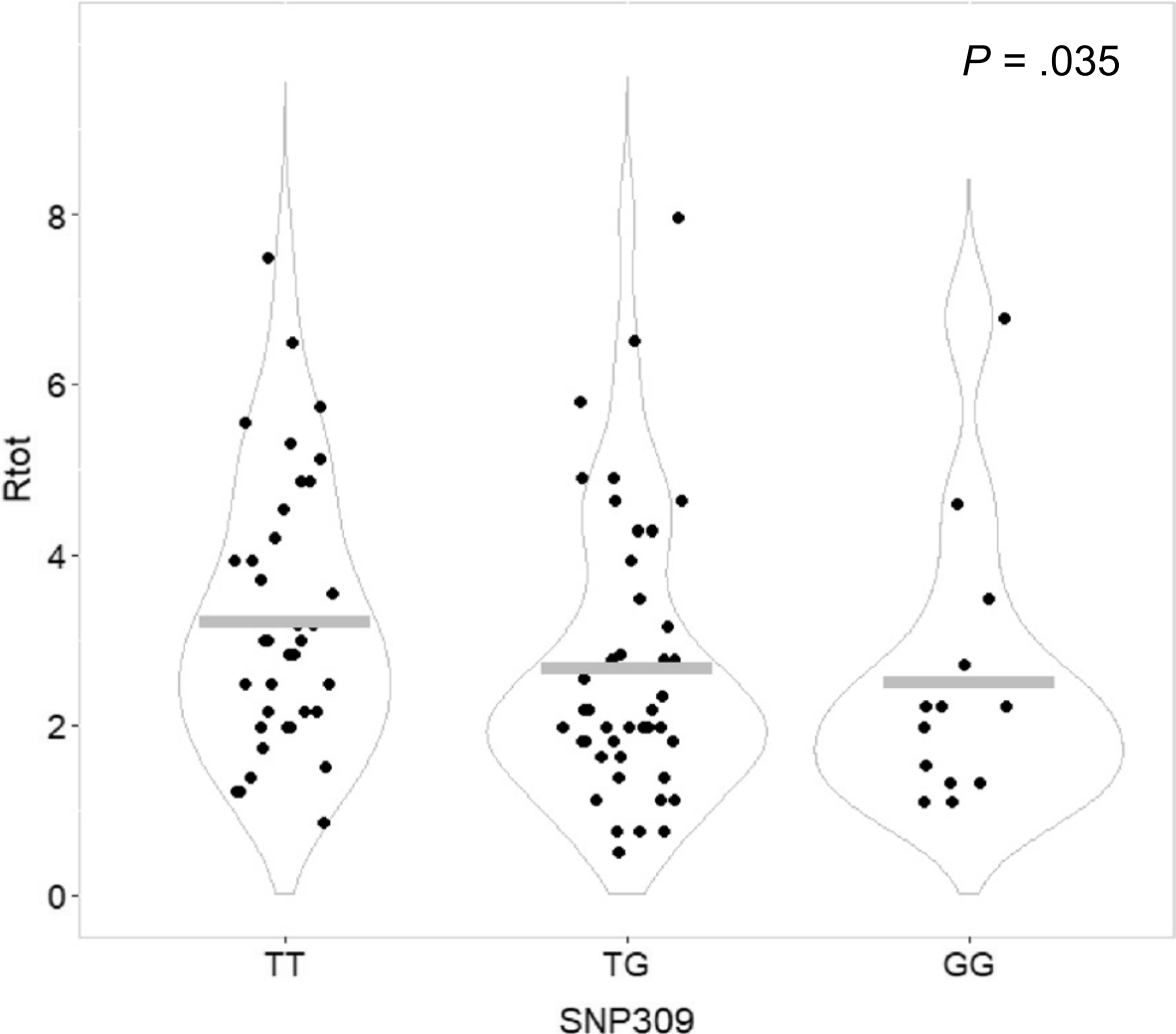
Neutrophil counts post-treatment/pre-treatment with neoadjuvant chemotherapy (R_tot_), stratified by *MDM2* SNP309 genotypes. Dot plot illustrating ratios of neutrophil counts post-treatment/pre-treatment (R_tot_) for breast cancer patients undergoing neoadjuvant chemotherapy, stratified by *MDM2* SNP309 genotypes. Grey bars indicate mean value in each group. P-value calculated by Jonkheere-Terpstra test for ranked groups (TT vs. TG vs. GG)

Assessing the ratios of neutrophil counts across the three genotypes of *MDM2* SNP309 (rs2279744), we found a significant association linking carriers of the G-allele to lower R_tot_ of neutrophils through the course of neoadjuvant treatment, as compared to those carrying the T-allele (R_tot_ *p* = 0.035; Table 1, Fig. 2). Further, assessing the *MDM2* SNP309 genotypes in the dominant model, we found the TT genotype to be favorable (higher R_tot_; *p* = 0.032), while one or two copies of the G-allele was related to lower R_tot_ of neutrophils (Table 1). Significance was lost in the recessive model.

**Table 1:** Impact of *MDM2* SNP309, *MDM2* SNP285 and *MDM2* del1518 status on neutrophil counts during chemotherapy

**Groups**	**Ratios**	**Neutrophils**
**(test)**		**(p)**
**SNP309**		
TT vs. TG vs. GG	R_epi_	0.942
(Jonckheere-Terpstra)	R_doc_	0.187
	R_tot_	**0.035**
TT vs. TG + GG	R_epi_	0.966
(Mann–Whitney)	R_doc_	0.206
	R_tot_	**0.032**
TT + TG vs. GG	R_epi_	0.844
(Mann–Whitney)	R_doc_	0.473
	R_tot_	0.298
**SNP285**		
GC vs. GG	R_epi_	0.325
(Mann–Whitney)	R_doc_	0.640
	R_tot_	0.413
**del1518**		
ii vs. id vs. dd	R_epi_	0.342
(Jonckheere-Terpstra)	R_doc_	0.521
	R_tot_	**0.049**
ii vs. id + dd	R_epi_	0.313
(Mann–Whitney)	R_doc_	0.605
	R_tot_	0.218
ii + id vs. dd	R_epi_	0.671
(Mann–Whitney)	R_doc_	0.555
	R_tot_	**0.036**

R_epi_ and R_doc_ represent the ratio of cell counts pre and post treatment with epirubicin and docetaxel respectively, while R_tot_ represents the ratio of cell counts before and after completing all courses of chemotherapy

ii: homozygous insertion genotype

id: heterozygous genotype

dd: homozygous deletion genotype

Having observed that the SNP309TG and GG genotypes were associated with a lower R_tot_ and the SNP309TT genotype to be associated with higher R_tot_, we explored whether this was specifically related to the epirubicin or the docetaxel treatment. Assessing R_epi_ of neutrophils, no significant differences were observed between individuals harboring the SNP309TT, SNP309TG or SNP309GG genotypes (R_epi_ *p* = 0.942; Table 1). Similarly, no associations were seen in the dominant or recessive models (R_epi_ *p* = 0.966 and 0.844, respectively). Neither were any significant associations detected for R_doc_ in the overall assessment nor in the dominant or recessive models (R_doc_ *p* = 0.187, 0.206 and 0.473, respectively). This indicated that the impact of *MDM2* SNP309 on the ratio of neutrophils was not specifically linked to either epirubicin or docetaxel, but rather an effect on the entire neoadjuvant treatment.

When applying the secondary measures, ratios of thrombocyte counts and hemoglobin values, we did not find any significant correlations across the three genotypes of *MDM2* SNP309 (R_tot_ *p* = 0.833 and 0.475, respectively; Supplementary Table 7). Notably, when analyzing total leukocyte count, our findings were very similar to those seen for neutrophil count: all associations detected when assessing neutrophils, were replicated when assessing total leukocytes, with p-values closely resembling those calculated based on neutrophils (Table 1; Supplementary Table 7).

### *MDM2* SNP285 status and neutrophil count during chemotherapy

For SNP285 (rs117039649), only genotypes GG (*n* = 98) and GC (*n* = 10) were represented in our dataset (Supplementary Table 2). No significant differences were observed when comparing either R_tot_, R_epi_ or R_doc_ for neutrophils between patients with these two genotypes. Neither were any significant differences observed when applying thrombocyte counts, hemoglobin values or total leukocyte counts as basis for the ratios (Table 1; Supplementary Table 7).

### *MDM2* del1518 status and neutrophil count during chemotherapy

**Fig. 3:**
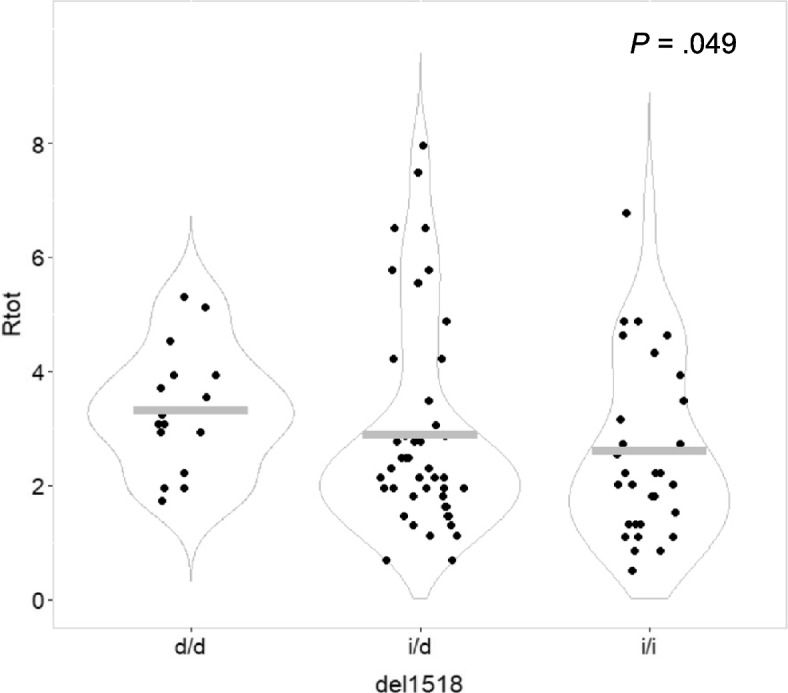
Neutrophil counts post-treatment/pre-treatment with neoadjuvant chemotherapy (R_tot_), stratified by *MDM2* del1518 genotypes. Dot plot illustrating ratios of neutrophil counts post-treatment/pre-treatment (R_tot_) for breast cancer patients undergoing neoadjuvant chemotherapy, stratified by *MDM2* del1518 genotypes. d/d = homozygous deletion genotype, i/d = heterozygous genotype, i/i = homozygous insertion type. Grey bars indicate mean value in each group. P-value calculated by Jonkheere-Terpstra test for ranked groups (d/d vs. i/d vs. i/i)

Assessing the ratio of neutrophils after sequential treatments vs. before treatment start (R_tot_) across the three different genotypes of the del1518 variant (rs3730485), we found patients harboring the del-allele to have a higher R_tot_, indicating better neutrophil recovery as compared to those with the ins-allele (higher R_tot_; *p* = 0.049; Table 1; Fig. 3).

The association was not retained as significant in a dominant model (*p* = 0.218) but in the recessive model we found a significantly higher R_tot_ among patients with the del/del genotype as compared to those with the ins/ins- or ins/del genotype (*p* = 0.036; Table 1).

Regarding the two treatments assessed separately, no significant differences were observed across the del1518 genotypes for R_epi_ and R_doc_ (Table 1), indicating that the impact of this polymorphism, similarly to SNP309, was likely related to the entire neoadjuvant treatment period.

Similarly, as for SNP309, when switching our analyses of del1518 to the secondary measures, ratios of thrombocyte counts and hemoglobin values, no associations were replicated when assessing the treatment as a whole (R_tot_ *p* = 0.932 and 0.165, respectively; Supplementary Table 7). In analyses for effect of treatment with epirubicin or docetaxel assessed separately, we found the del1518 del/del genotype to be associated with a lower R_doc_ of hemoglobin in the recessive model (R_doc_ *p* = 0.038; Supplementary Table 7), in contrast with the higher R_tot_ observed when assessing the main measure of neutrophils. The associations detected when assessing neutrophils, were again replicated when assessing total leukocytes, with p-values very close to those calculated based on neutrophils (Table 1; Supplementary Table 7).

### Combinatorial genotypes

**Fig. 4:**
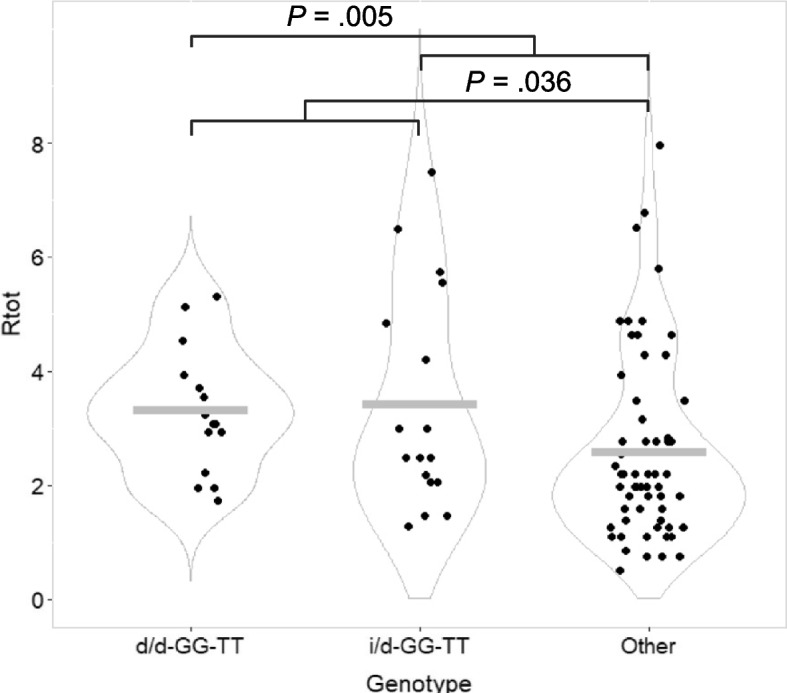
Neutrophil counts post-treatment/pre-treatment with neoadjuvant chemotherapy (R_tot_), stratified by combinatorial *MDM2* polymorphism genotypes. Dot plot illustrating ratios of neutrophil counts post-treatment/pre-treatment (R_tot_) for breast cancer patients undergoing neoadjuvant chemotherapy, stratified by combinatorial *MDM2* polymorphism genotypes. d/d-GG-TT = patients with del1518 deletion/deletion, SNP285GG and SNP309TT genotypes. i/d-GG-TT = patients with del1518 insertion/deletion, SNP285GG and SNP309TT genotypes. Grey bars indicate mean value in each group. P-value calculated by Mann–Whitney tests for two groups

Given the observations of the *MDM2* SNP309T- and the del1518 del-allele to have a favorable impact on neutrophil levels throughout chemotherapy treatment, we assessed whether combinatorial genotypes with both alleles present had a particularly high impact on neutrophil counts. An overview of the R_tot_ for the different genotypes is given in Supplementary Fig. 3. We found patients carrying genotype SNP309TT / del1518 del-del to have a significantly higher R_tot_ than patients harboring the remaining genotypes (*p* = 0.036; Table 2; Fig. 4). This association was not replicated in our secondary measures (Supplementary Table 8).

**Table 2:** Combinatorial genotypes of *MDM2* polymorphisms and impact on neutrophil counts

Groups	Ratios	Neutrophils
**(test)**		**(p)**
SNP309TT-del1518dd vs. Rest	R_epi_	0.671
(Mann–Whitney)	R_doc_	0.555
	R_tot_	**0.036**
SNP309TT-del1518id vs. Rest	R_epi_	0.601
(Mann–Whitney)	R_doc_	0.321
	R_tot_	0.164
SNP309TT-del1518dd + id vs. Rest	R_epi_	0.442
(Mann–Whitney)	R_doc_	0.206
	R_tot_	**0.005**

R_epi_ and R_doc_ represent the ratio of cell counts pre and post treatment with epirubicin and docetaxel respectively, while R_tot_ represents the ratio of cell counts before and after completing all courses of chemotherapy

ii: homozygous insertion genotype

id: heterozygous genotype

dd: homozygous deletion genotype

Further, adding the patients with SNP309TT and del1518 ins-del, to the test group, we found that these individuals (with genotype SNP309TT and del1518 del-del or ins-del) had a particularly well sustained neutrophil count throughout the treatment as compared to the rest (R_tot_ *p* = 0.005; Table 2; Fig. 4).

Assessing the same combinatorial genotypes of SNP309 and del1518 with respect to R_epi_ and R_doc_ of neutrophils, no significant differences were observed (Table 2).

SNP285 has an even stronger linkage disequilibrium to SNP309 than the del1518 polymorphism, due to the very close proximity of the two SNPs (24 bp). However, the low number of SNP285 genotype GC (*n* = 10) and CC (*n* = 0) precluded formal statistical assessment of combinatorial genotypes between this SNP and SNP309. Since the SNP285C-allele has been found to functionally counteract the effects of SNP309 [16], we re-analyzed our data for SNP309-associations, removing the 10 patients harboring the SNP285GC genotype. Doing so had only a minor impact on the associations (Supplementary Table 9). Further, since SNP285C only exists on the SNP309G-allele, we also re-assessed the SNP285 calculations, excluding patients with SNP309TT genotype. We did not observe any significant associations in these assessments (Supplementary Table 10).

### Baseline similarity

Given the observed differences in ratios of neutrophils related to SNP309 and del1518, and combinatorial genotypes of the two, we went on to assess whether these were mainly due to differences acquired during the treatment with chemotherapy and G-CSF prophylaxis, or if they simply reflect differences in the baseline (pretreatment) levels of neutrophils. We found no significant difference in pretreatment neutrophil counts across genotypes SNP309, del1518, or combinatorial genotypes. Patients harboring the SNP309TT / del1518 del-del or ins-del genotype had a significantly higher post-treatment neutrophil counts (Supplementary Table 11; Supplementary Fig. 2a), thus explaining differences observed when assessing ratios of neutrophils.

## Discussion

The p53 protein plays a key role in regulating vital cellular functions including apoptosis, growth arrest, DNA repair and several additional functions [32]. Acting as a major regulator of p53 activity [5], *MDM2* and functional polymorphisms in its promoter have received attention with respect to risk for different cancer forms (for review see [33]). In general, the SNP309 G-allele has been linked to increased cancer risk and the SNP285 C-allele has been linked to reduced cancer risk, while the results for del1518 have been less conclusive, but there are clear indications that the effect of the different polymorphisms may be tissue-dependent [34].

Although the main focus has been on cancer risk, there have also been numerous studies assessing the impact of *MDM2* polymorphisms on non-malignant phenotypes. Thus, the SNP309 has been linked to e.g. trisomy 21 [35], fertility [36], missed abortion [37] and rheumatoid arthritis [23, 38].

The genotype distribution of the *MDM2* polymorphisms varies greatly between populations [29]. We previously identified a clear west–east gradient of SNP285C, with higher MAFs in Europe, and an inverse gradient for SNP309. Others have investigated frequency differences of *MDM2* SNPs and other polymorphisms in the *MDM2-TP53* axis with respect to latitude [39] and climate variables [25], but the results are not conclusive. Jacovas and colleagues provided an interesting contribution to this discussion when reporting that there seemed to be an evolutionary selection for the SNP309TT genotype in populations living at high altitudes [24], potentially indicating a role in hematopoiesis.

In the present study, we found the SNP309 T-allele and the del1518 del-allele to be associated with better sustained neutrophil counts during dose-dense neoadjuvant chemotherapy in primary breast cancer patients. Notably, assessing our secondary measures of thrombocyte counts and hemoglobin levels did not reproduce a similar association, indicating that the impact of *MDM2* polymorphisms may be especially related to neutrophils, rather than hematopoietic recovery in general.

To the best of our knowledge, only one previous study has addressed the potential impact of *MDM2* polymorphisms on neutrophil levels during chemotherapy in breast cancer. Okishiro and colleagues found the SNP309GG genotype to be linked to a lower incidence of severe neutropenia in patients receiving the highest dose ranges of a combinatorial 5FU/epirubicin/cyclophosphamide (FEC) regimen [26]. Although not directly comparable due to different cancer forms and chemotherapy regimens, Wang and colleagues reported similar findings, with lower incidences of grade III/IV neutropenia among patients harboring the SNP309GG in a cohort of small-cell lung cancer patients receiving a combination of etoposide and cisplatin [40]. While this may seem contradictory to our present results, the chemotherapy regimens were different with 5FU, cyclophosphamide and epirubicin administered in concert to the Japanese patients [26] and etoposide and cisplatin to the Chinese [40], while our present (Norwegian) patients had epirubicin and docetaxel, both administered sequentially as monotherapy. Second, the measures used for bone marrow function were different (severe neutropenia versus ratios of neutrophil counts). Third, and probably most importantly, all patients in our DDP-trial received subcutaneous pegfilgrastim (G-CSF) 6 mg injection 24 h after each chemotherapy cycle (primary prophylaxis), while in the studies reported by Okishiro and Wang and their colleagues, only patients experiencing febrile neutropenia received G-CSF treatment on subsequent cycles. Therefore, since all our patients received G-CSF, the associations seen in the present study may relate to some hitherto unknown impact of *MDM2*-polymorphisms on pharmacological parameters for the G-CSF treatment, rather than directly on neutrophils and bone marrow recovery. Notably, we observed high ratios of R_tot_, and many patients had ratios > 1,0 indicating an increase in neutrophil count compared to samples taken before neoadjuvant chemotherapy (Supplementary Table 3). Finally, it should also be noted that there are differences regarding the *MDM2* genotype distribution between the populations [29].

Given the strong linkage disequilibrium between the *MDM2* promoter polymorphisms, it may not be optimal to assess individual polymorphism without taking the haplotype structure into account. As such, a strength of the present study was that we did not only assess single polymorphisms but also assessed specific genotype combinations. Doing so, we found the SNP309TT in combination with at least one del1518 del-allele to be associated with significantly higher R_tot_ of neutrophils. Hence, we identified a subgroup of patients with particularly well sustained neutrophil levels during chemotherapy treatment, beyond what could be done by assessment of single polymorphisms. Even if these findings are encouraging and provide new insight into the role of MDM2, the difference between patients with the different genotypes seems too limited for clinical use as biomarkers for G-CSF. Clinical use would perhaps require that this information is merged with other relevant data, e.g. in a composite (polygenic) biomarker profile.

## Conclusion

We found the *MDM2* polymorphisms SNP309 and del1518 to be associated with neutrophil recovery during neoadjuvant chemotherapy in breast cancer patients. The SNP309 reference T-allele and the alternative del-allele of the del1518 were associated with a better sustained neutrophil count. In combined genotype-analyses, patients harboring the SNP309 TT genotype and at least one copy of the del1518 del-allele had particularly favorably sustained neutrophil counts.

## Supplementary Information

Supplementary Material 1.

## Data Availability

The datasets generated and analyzed in the current study are available via the FigShare repository (doi:10.6084/m9.figshare.27931146, doi:10.6084/m9.figshare.28211267) and in the Supplementary Information (Supplementary Table 3). Upon formal request, further de-identified information about the sample donors, may be shared according to institutional guidelines, pending project-specific approvals from the Regional Ethics Committee in Norway. Requests are via a standard pro forma describing the nature of the proposed research and extent of data requirements. Data recipients are required to enter a formal data sharing agreement that describes the conditions for release and requirements for data transfer, storage, archiving, publication, and intellectual property. Requests are reviewed by the DDP study team in terms of scientific merit and ethical considerations, including patient consent. Requests may be directed to the corresponding author.

## Abbreviations

FEC: Combinatorial 5FU/epirubicin/cyclophosphamide regimen
G-CSF: Granulocyte colony stimulating factor
R_tot_: Ratio of neutrophil counts post vs. pre-treatment with epirubicin and docetaxel treatment
R_epi_: Ratio of neutrophil counts post vs. pre-treatment with epirubicin
R_doc_: Ratio of neutrophil counts post vs. pre-treatment with docetaxel
