# Supplementary material for: The impact of functional MDM2-polymorphisms on neutrophil counts in breast cancer patients during neoadjuvant chemotherapy
Source: BMC Cancer. 2025 Feb 20;25:308. doi: 10.1186/s12885-025-13675-2 (PMC11843751; doi:10.1186/s12885-025-13675-2)
Supplement: Supplementary file 1 — Supplementary Material 1. [file 12885_2025_13675_MOESM1_ESM.pdf]

Supplementary Information

Supplementary Fig. 1: Flow diagram of patients included in the present study (Dose Dense Protocol, DDP), illustrating treatment given and the ratios of neutrophil counts included in statistical analyses (R_{epi} , R_{doc}, R_{tot}). Epicubicin and docetaxel was administered q2w, details provided in the methods section. Blood samples including differential leukocyte count were drawn prior to each chemotherapy cycle, numbered 1 to 8. Ratio during epirubicin treatment was calculated as ratio of values from blood sample 5 divided by values from blood sample 1 (R_{epi}). Similarly, the ratio for treatment with docetaxel (R_{doc}) was defined as sample 8/sample 5 while the ratio for the complete treatment (R_{tot}) was defined as sample 8/sample 1.

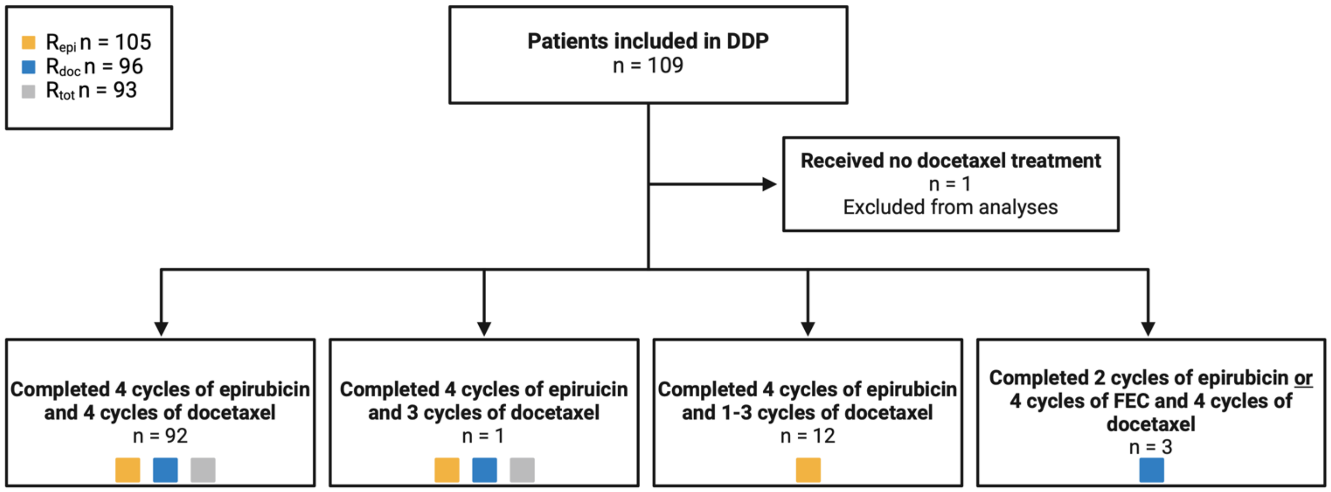

Supplementary Table 1: Patients not completing the total neoadjuvant treatment of epirubicin (Epi) and docetaxel (Doc), or who received a different regimen (FEC) due to suspected metastasis, therefore only included in the present study for calculations of R_{epi} or R_{doc}, not R_{tot}. Blood samples including differential leukocyte count were drawn prior to each chemotherapy cycle (Course 1 to 8). R_{epi} and R_{doc} represent the ratio of neutrophil cell counts pre and post treatment with epirubicin and docetaxel respectively.

StudyID	Course 1	Course 2	Course 3	Course 4	Course 5	Course 6	Course 7	Course 8	Included ratios
DDP 03	Epi	Epi	Doc	Doc	Doc	Doc	Doc		R_{doc} (3-6)
DDP 04	Epi	Epi	Epi	Epi	Doc	Doc	Doc		R_{epi} (1-5)
DDP 10	Epi	Epi	Epi	Epi	Doc	Doc			R_{epi} (1-5)
DDP 17	Epi	Epi	Doc	Doc	Doc	Doc			R_{doc} (3-6)
DDP 33	Epi	Epi	Epi	Epi	Doc	Doc			R_{epi} (1-5)
DDP 35	Epi	Epi	Epi	Epi	Doc	Doc			R_{epi} (1-5)
DDP 36	Epi	Epi	Epi	Epi	Doc	Doc	Doc		R_{epi} (1-5)
DDP 39	Epi	Epi	Epi	Epi	Doc	Doc	Epi	Epi	R_{epi} (1-5)
DDP 67	Epi	Epi	Epi	Epi	Doc	Doc	Epi	Epi	R_{epi} (1-5)
DDP 78	FEC	FEC	FEC	FEC	Doc	Doc	Doc	Doc	R_{doc} (5-8)
DDP 79	Epi	Epi	Epi	Epi	Doc	Doc			R_{epi} (1-5)
DDP 83	Epi	Epi	Epi						-
DDP 87	Epi	Epi	Epi	Epi	Doc	Doc	Doc		R_{epi} (1-5)
DDP 90*	Epi	Epi	Epi	Epi	Doc	Doc	Doc		R_{epi} (1-5) R_{doc} (5-8)
DDP 106	Epi	Epi	Epi	Epi	Doc	Doc (weekly)			R_{epi} (1-5)
DDP 107	Epi	Epi	Epi	Epi	Doc	Doc	Doc	Doc (weekly)	R_{epi} (1-5)
DDP 108	Epi	Epi	Epi	Epi	Doc	Doc (weekly)			R_{epi} (1-5)

*DDP 90 did not complete course 8, however, blood sample was drawn prior to the planned eighth course and the patient could therefore be included in R_{tot} calculations.

Supplementary Table 2: Genotype distribution of *MDM2* promoter polymorphisms.

***MDM2* polymorphism**	**Genotype**			**MAF**	**H-W (p)**
SNP309 (rs2279744)	TT	TG	GG		
n (%)	46 (42.6%)	47 (43.5%)	15 (13.9%)	0.36	0.87
SNP285 (rs117039649)	GG	GC	CC		
n (%)	98 (90.7%)	10 (9.3%)	0 (0.0%)	0.05	0.88
del1518 (rs3730485)	ins/ins	ins/del	del/del		
n (%)	37 (34.3%)	52 (48.1%)	19 (17.6%)	0.37	0.99

MAF: Minor allele frequency
H-W (p): p-value for Hardy-Weinberg equilibrium

Supplementary Table 3: *MDM2* polymorphism status and calculated ratios for all patients included in the present study, here sorted by SNP309 genotype, then by del1518 and SNP285 genotype. R_{epi} and R_{doc} represent the ratio of cell counts pre and post treatment with epirubicin and docetaxel respectively, while R_{tot} represents the ratio of cell counts before and after completing all courses of chemotherapy.

**StudyID**	**SNP309**	**del1518**	**SNP285**	**Neutrophils**			**Leukocytes**			**Thrombocytes**			**Hemoglobin**		
				R_{doc}	R_{epi}	R_{tot}	R_{doc}	R_{epi}	R_{tot}	R_{doc}	R_{epi}	R_{tot}	R_{doc}	R_{epi}	R_{tot}
DDP 03	TT	d/d	GG	0.97			0.95			0.45			0.87		
DDP 09	TT	d/d	GG	1.39	2.32	3.24	1.17	1.92	2.25	0.89	0.77	0.68	0.85	0.95	0.81
DDP 20	TT	d/d	GG	1.61	3.19	5.12	1.47	2.36	3.47	0.88	1.12	0.98	0.85	0.94	0.80
DDP 26	TT	d/d	GG	4.72	0.75	3.54	3.72	0.87	3.22	0.93	1.00	0.93	0.96	0.83	0.80
DDP 33	TT	d/d	GG		1.04			1.01			0.95			0.81	
DDP 40	TT	d/d	GG	1.59	1.90	3.03	1.29	1.72	2.21	0.77	0.98	0.76	0.88	0.89	0.78
DDP 41	TT	d/d	GG	1.30	1.50	1.95	1.16	1.32	1.53	1.05	0.89	0.93	0.86	0.91	0.78
DDP 43	TT	d/d	GG	1.38	3.85	5.31	1.10	2.55	2.81	1.06	0.90	0.95	0.88	0.85	0.75
DDP 48	TT	d/d	GG	1.52	1.45	2.21	1.29	1.29	1.66	1.25	0.99	1.24	0.94	0.97	0.91
DDP 50	TT	d/d	GG	3.25	1.21	3.93	2.64	1.17	3.09	1.05	1.13	1.19	0.87	0.95	0.83
DDP 52	TT	d/d	GG	2.45	1.22	3.00	2.23	1.15	2.57	0.82	0.66	0.54	0.86	0.87	0.75
DDP 53	TT	d/d	GG	0.97	3.83	3.71	0.95	3.13	2.96	1.48	0.60	0.89	0.87	1.01	0.88
DDP 59	TT	d/d	GG	1.69	1.71	2.88	1.47	1.21	1.79	0.97	1.02	0.98	0.95	0.86	0.82
DDP 62	TT	d/d	GG	1.20	1.63	1.95	1.20	1.33	1.59	0.83	0.72	0.59	0.88	0.91	0.79
DDP 67	TT	d/d	GG		1.39			1.23			1.13			0.87	
DDP 77	TT	d/d	GG	1.19	2.61	3.11	1.09	2.03	2.22	0.90			0.95	0.86	0.82
DDP 84	TT	d/d	GG	1.85	2.45	4.53	1.59	1.74	2.77	1.20	0.93	1.12	0.88	0.86	0.76
DDP 89	TT	d/d	GG	2.47	0.70	1.73	2.42	0.73	1.76	0.45	1.11	0.50	0.84	0.88	0.74
DDP 102	TT	d/d	GG	3.95	1.00	3.95	2.79	1.10	3.07	0.85	0.94	0.80	0.96	0.90	0.86
DDP 04	TT	d/i	GG		3.06			2.67			1.62			0.82	
DDP 12	TT	d/i	GG	1.42	1.42	2.01	1.30	1.30	1.70	0.79	0.99	0.78	0.89	0.93	0.83
DDP 14	TT	d/i	GG	4.93	0.98	4.83	3.00	1.01	3.04	0.67	1.16	0.77	0.96	0.87	0.84
DDP 24	TT	d/i	GG	1.22	1.17	1.43	1.09	1.03	1.12	0.86	0.67	0.58	0.89	0.86	0.76
DDP 25	TT	d/i	GG	1.99	2.79	5.55	1.74	2.22	3.86	0.56	1.14	0.64	1.05	0.94	0.99
DDP 35	TT	d/i	GG		2.20			1.37			1.06			0.97	

DDP 42	TT	d/i	GG	1.08	2.28	2.47	1.03	1.95	2.00	1.01	1.04	1.05	0.89	0.92	0.83
DDP 54	TT	d/i	GG	1.42	1.06	1.51	1.26	1.08	1.37	1.58	0.73	1.16	0.89	0.87	0.77
DDP 60	TT	d/i	GG	2.46	1.71	4.21	2.04	1.50	3.07	1.09	0.96	1.05	0.98	0.88	0.87
DDP 70	TT	d/i	GG	1.01	2.44	2.47	1.10	1.71	1.89	1.45	0.70	1.02	0.90	0.90	0.80
DDP 79	TT	d/i	GG		1.93			1.55			1.90			0.87	
DDP 87	TT	d/i	GG		4.77			2.69			1.05			0.87	
DDP 88	TT	d/i	GG	6.35	1.02	6.50	4.57	1.19	5.42	0.78	1.16	0.90	0.89	0.87	0.78
DDP 92	TT	d/i	GG	1.79	1.39	2.49	1.57	1.33	2.09	1.18	0.81	0.96	0.98	0.88	0.86
DDP 97	TT	d/i	GG	1.09	2.80	3.06	0.97	2.06	2.00	0.94	0.88	0.83	0.92	0.84	0.78
DDP 99	TT	d/i	GG	0.88	1.46	1.28	0.81	1.49	1.21	1.02	0.79	0.80	0.87	0.90	0.78
DDP 100	TT	d/i	GG	1.69	1.24	2.10	1.31	1.29	1.70	0.70	1.19	0.83	0.93	0.89	0.83
DDP 104	TT	d/i	GG	6.54	1.15	7.50	4.97	1.11	5.49	0.93	1.00	0.93	0.92	0.94	0.87
DDP 105	TT	d/i	GG	2.10	2.74	5.74	1.91	1.81	3.45	0.95	1.10	1.05	1.06	0.81	0.86
DDP 107	TT	d/i	GG		1.32			1.15			1.24			0.84	
DDP 115	TT	d/i	GG	1.41	1.55	2.18	1.23	1.36	1.67	0.93	0.87	0.81	0.88	0.87	0.76
DDP 116	TT	d/i	GG	3.30	0.88	2.92	1.80	1.24	2.24	1.28	1.21	1.55	0.88	0.97	0.85
DDP 34	TT	i/i	GG	3.97	1.23	4.87	2.78	1.18	3.29	0.80	0.99	0.79	0.99	0.97	0.96
DDP 65	TT	i/i	GG	0.57	1.50	0.85	0.63	1.24	0.78	1.87	1.01	1.89	0.96	0.82	0.79
DDP 72	TT	i/i	GG	1.83	0.63	1.15	1.38	0.79	1.10	0.77	0.94	0.72	0.91	0.89	0.81
DDP 80	TT	i/i	GG	2.77	1.00	2.77	2.01	1.12	2.25	1.31	0.70	0.91	0.82	0.95	0.78
DDP 103	TT	i/i	GG	0.92	1.42	1.31	0.71	1.30	0.92	1.04	1.34	1.39	0.86	0.81	0.70
DDP 01	TG	d/i	GG	1.89	1.42	2.69	1.85	1.34	2.47	0.79	1.24	0.97	1.07	0.78	0.83
DDP 11	TG	d/i	GG	3.03	2.15	6.52	2.52	1.72	4.34	0.85	0.77	0.66	0.89	0.97	0.86
DDP 13	TG	d/i	GG	2.28	2.54	5.80	1.87	1.91	3.57	0.80	1.23	0.99	0.96	0.93	0.89
DDP 19	TG	d/i	GG	1.07	1.64	1.76	1.06	1.46	1.54	0.92	0.81	0.75	0.91	0.95	0.87
DDP 28	TG	d/i	GG	3.20	0.89	2.84	2.28	1.03	2.34	1.13	1.01	1.14	0.93	0.87	0.81
DDP 36	TG	d/i	GG		1.73			1.29			1.04			0.81	
DDP 37	TG	d/i	GG	1.09	1.80	1.95	0.98	1.62	1.59	1.30	0.87	1.13	0.92	0.83	0.76
DDP 44	TG	d/i	GG	2.07	1.32	2.74	1.41	1.21	1.71	1.21	0.99	1.21	0.88	0.84	0.74
DDP 46	TG	d/i	GG	0.21	3.18	0.68	0.31	2.83	0.87	1.22	1.31	1.60	0.87	0.88	0.77
DDP 47	TG	d/i	GG	1.44	1.62	2.34	1.32	1.41	1.86	1.08	0.78	0.84	0.95	0.84	0.80
DDP 51	TG	d/i	GG	1.84	0.90	1.65	1.70	0.81	1.39	1.09	0.92	1.00	0.93	0.89	0.82
DDP 58	TG	d/i	GG	1.71	1.24	2.12	1.61	1.22	1.96	1.11	0.91	1.01	0.84	0.93	0.78
DDP 61	TG	d/i	GG	0.50	2.63	1.33	0.55	2.09	1.16	0.86	1.02	0.87	0.87	0.96	0.84
DDP 66	TG	d/i	GG	2.89	0.75	2.17	1.59	1.07	1.70	0.90	0.93	0.84	0.86	0.90	0.77
DDP 81	TG	d/i	GG	0.81	1.43	1.16	0.81	1.25	1.01	1.04	0.91	0.95	0.91	0.92	0.84
DDP 82	TG	d/i	GG	0.96	2.94	2.83	0.93	2.09	1.94	0.76	1.14	0.86	0.87	0.92	0.80
DDP 86	TG	d/i	GG	1.00	1.43	1.43	0.99	1.06	1.04	0.90	0.95	0.86	0.92	0.88	0.80
DDP 90	TG	d/i	GG	3.33	2.39	7.97	2.78	1.85	5.16	1.52	1.01	1.53	0.88	0.87	0.76
DDP 91	TG	d/i	GG	1.83	1.02	1.86	1.62	1.04	1.68	1.13	1.15	1.30	0.97	0.89	0.86
DDP 94	TG	d/i	GG	3.11	1.58	4.90	2.43	1.43	3.47	1.26	0.92	1.16	0.89	0.85	0.76
DDP 95	TG	d/i	GG	1.33	3.18	4.23	1.31	2.24	2.93	1.12	1.08	1.21	0.91	0.87	0.79
DDP 96	TG	d/i	GG	0.91	3.85	3.49	0.85	3.22	2.73	1.25	0.95	1.19	1.04	0.79	0.82
DDP 112	TG	d/i	GG	0.50	2.11	1.05	0.56	1.94	1.09	0.99	0.68	0.68	0.81	0.91	0.73
DDP 114	TG	d/i	GG	3.00	0.65	1.95	2.58	0.71	1.84	1.36	0.85	1.15	0.90	0.84	0.75
DDP 117	TG	d/i	GG	0.26	2.62	0.69	0.29	1.81	0.53	0.86	1.07	0.92	0.98	0.86	0.84
DDP 16	TG	d/i	GC	1.20	1.59	1.91	1.10	1.49	1.64	0.86	1.21	1.04	1.00	0.83	0.83
DDP 74	TG	d/i	GC	1.30	1.73	2.25	1.29	1.44	1.86	0.89	0.88	0.78	0.86	0.89	0.76
DDP 98	TG	d/i	GC	2.94	0.68	2.01	2.46	0.73	1.79	0.84	1.09	0.91	0.92	0.89	0.82
DDP 101	TG	d/i	GC	1.11	1.07	1.19	0.93	1.20	1.11	0.79	0.95	0.76	0.89	0.87	0.77
DDP 110	TG	d/i	GC	1.94	0.82	1.59	1.64	0.93	1.52	1.21	0.91	1.10	0.86	0.89	0.76

DDP 15	TG	i/i	GG	3.64	1.26	4.59	2.96	1.20	3.57	0.88	1.48	1.30	0.91	0.94	0.86
DDP 21	TG	i/i	GG	0.84	2.24	1.88	0.79	1.56	1.24	0.95	0.79	0.75	0.91	0.95	0.86
DDP 27	TG	i/i	GG	1.17	2.17	2.54	1.12	1.89	2.13	0.92	0.96	0.88	0.85	0.95	0.81
DDP 30	TG	i/i	GG	1.10	1.86	2.05	1.35	1.44	1.95	0.98	1.05	1.03	1.03	0.84	0.87
DDP 31	TG	i/i	GG	0.51	1.63	0.83	0.62	1.40	0.87	2.22	1.01	2.23	0.97	0.87	0.84
DDP 32	TG	i/i	GG	2.14	0.81	1.74	1.57	0.95	1.48	1.38	0.89	1.22	1.02	0.94	0.95
DDP 39	TG	i/i	GG		1.31			1.37			1.03			0.90	
DDP 55	TG	i/i	GG	2.42	1.63	3.94	2.10	1.44	3.04	1.05	1.11	1.17	0.92	0.80	0.74
DDP 71	TG	i/i	GG	2.01	2.31	4.65	1.76	1.79	3.15	0.88	0.66	0.58	0.83	0.89	0.74
DDP 76	TG	i/i	GG	1.88	2.30	4.32	1.65	1.94	3.20	0.88	0.94	0.83	0.88	0.88	0.77
DDP 78	TG	i/i	GG	4.23			2.08			0.72			1.16		
DDP 85	TG	i/i	GG	2.10	0.95	1.99	1.70	0.94	1.59	0.87	1.17	1.02	0.90	0.91	0.82
DDP 108	TG	i/i	GG		1.16			1.05			0.96			0.92	
DDP 113	TG	i/i	GG	1.16	2.72	3.16	1.08	1.84	1.98	1.02	0.88	0.89	0.84	0.85	0.72
DDP 07	TG	i/i	GC	0.75	0.66	0.50	0.77	0.72	0.56	0.88	0.95	0.83	0.92	0.95	0.88
DDP 17	TG	i/i	GC	0.98			0.86			0.86			0.82		
DDP 75	TG	i/i	GC	2.53	1.94	4.90	2.30	1.29	2.96	1.15	0.88	1.01	0.93	0.87	0.80
DDP 02	GG	i/i	GG	2.11	1.28	2.70	1.94	1.12	2.17	0.87	1.00	0.87	0.85	0.93	0.79
DDP 08	GG	i/i	GG	0.91	1.49	1.35	0.97	1.41	1.38	0.90	0.86	0.77	1.18	0.96	1.13
DDP 10	GG	i/i	GG		1.88			1.42			1.04			0.90	
DDP 29	GG	i/i	GG	0.81	1.44	1.16	0.81	1.36	1.09	0.98	0.98	0.95	0.95	0.97	0.92
DDP 38	GG	i/i	GG	1.10	1.18	1.29	0.86	1.11	0.96	0.72	0.92	0.66	0.95		
DDP 45	GG	i/i	GG	1.34	1.62	2.17	1.19	1.38	1.64	1.17	0.95	1.11	0.89	0.92	0.82
DDP 56	GG	i/i	GG	0.47	2.20	1.03	0.55	1.69	0.93	0.72	0.99	0.71	1.05	0.80	0.84
DDP 63	GG	i/i	GG	1.70	0.89	1.52	1.49	0.87	1.30	0.88	0.74	0.65	0.84	0.90	0.75
DDP 64	GG	i/i	GG	2.07	2.22	4.59	1.66	1.64	2.72	1.18	0.87	1.02	0.98	0.87	0.85
DDP 73	GG	i/i	GG	2.88	1.21	3.48	2.31	1.19	2.75	0.96	0.91	0.87	0.92	0.89	0.82
DDP 93	GG	i/i	GG	1.92	1.17	2.24	1.70	1.04	1.78	1.26	0.72	0.91	0.86	0.91	0.78
DDP 106	GG	i/i	GG		1.72			1.42			1.34			0.73	
DDP 109	GG	i/i	GG	1.43	1.39	1.98	1.30	1.27	1.65	1.04	0.75	0.78	0.92	0.89	0.82
DDP 49	GG	i/i	GC	1.08	2.09	2.26	1.08	1.59	1.72	0.74	1.05	0.77	0.98	0.86	0.85
DDP 69	GG	i/i	GC	2.69	2.52	6.78	2.13	1.80	3.84	0.69	0.83	0.57	0.95	0.97	0.92

i/i: homozygous insertion genotype
d/i: heterozygous genotype
d/d: homozygous deletion genotype

Supplementary Table 4: Association between *MDM2* SNP309, *MDM2* SNP285 and *MDM2* del1518 and measures of Age and Body Mass Index (BMI), possible factors influencing on hematological parameters during chemotherapy.

Groups (test)	Age (p)	BMI (p)
**SNP309**		
TT vs. TG vs. GG (Jonckheere-Terpstra)	0.288	0.595
**SNP285**		
GG vs. GC (Mann-Whitney)	0.803	0.051
**Del1518**		
i/i vs. d/i vs. d/d (Jonckheere-Terpstra)	0.069	0.137

i/i: homozygous insertion genotype
d/i: heterozygous genotype
d/d: homozygous deletion genotype

Supplementary Table 5: Correlation between ratios and measures of Age and BMI, possible factors influencing on hematological parameters during chemotherapy. R_{epi} and R_{doc} represent the ratio of cell counts pre and post treatment with epirubicin and docetaxel respectively, while R_{tot} represents the ratio of cell counts before and after completing all courses of chemotherapy.

Groups (test)	Ratios	Neutrophils (p)	Leukocytes (p)	Thrombocytes (p)	Hemoglobin (p)
Correlation Age and Ratios (Spearman's Rho)	R_{epi}	0.071	0.083	0.054	**0.033**
	R_{doc}	0.204	0.347	0.196	0.360
	R_{tot}	0.106	0.125	**0.019**	0.706
Correlation BMI and Ratios (Spearman's Rho)	R_{epi}	0.814	0.808	**<0.001**	0.144
	R_{doc}	0.559	0.538	0.773	0.302
	R_{tot}	0.451	0.419	**0.015**	0.833

Supplementary Table 6: Separate file containing total leukocyte counts, neutrophil counts, thrombocytes and hemoglobin values, and all calculations used in statistical analysis, available at FigShare (doi: 10.6084/m9.figshare.28211267).

Supplementary Fig. 2 A Line chart illustrating the development of neutrophil counts during chemotherapy treatment, with pre treatment values at cycle 1, grouped by SNP309 genotypes, **B** Line chart illustrating the development of ratios of neutrophil counts during chemotherapy treatment, with pre treatment values at cycle 1, grouped by SNP309 genotypes.

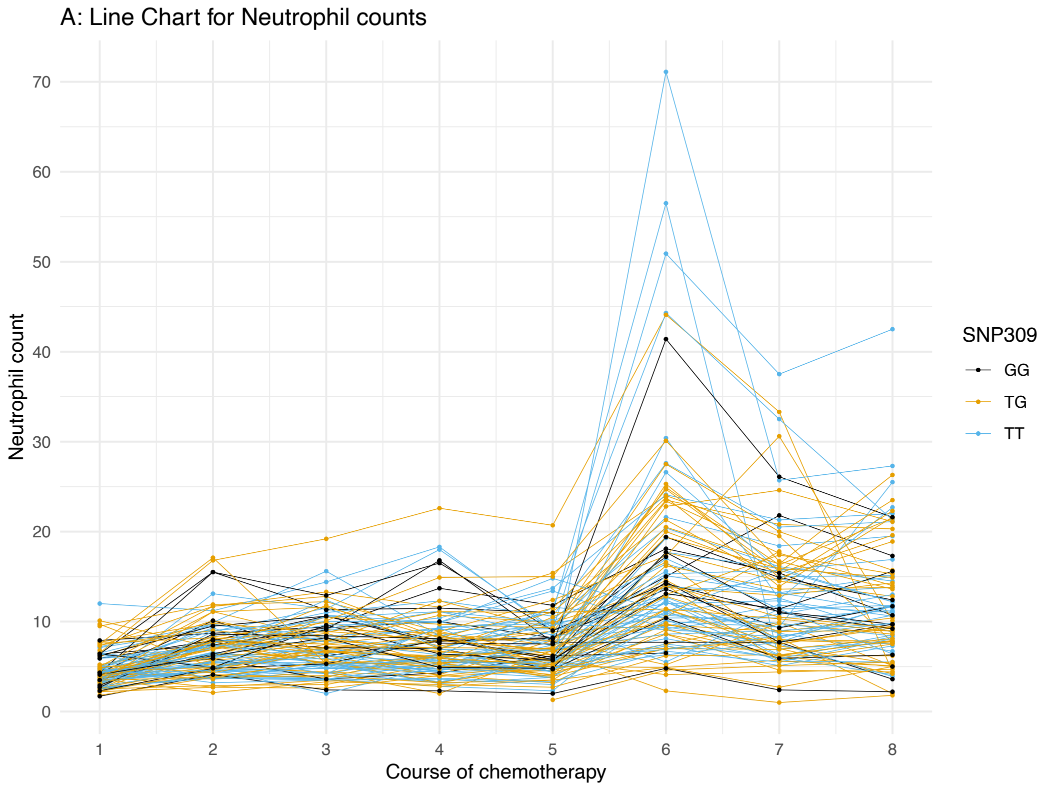

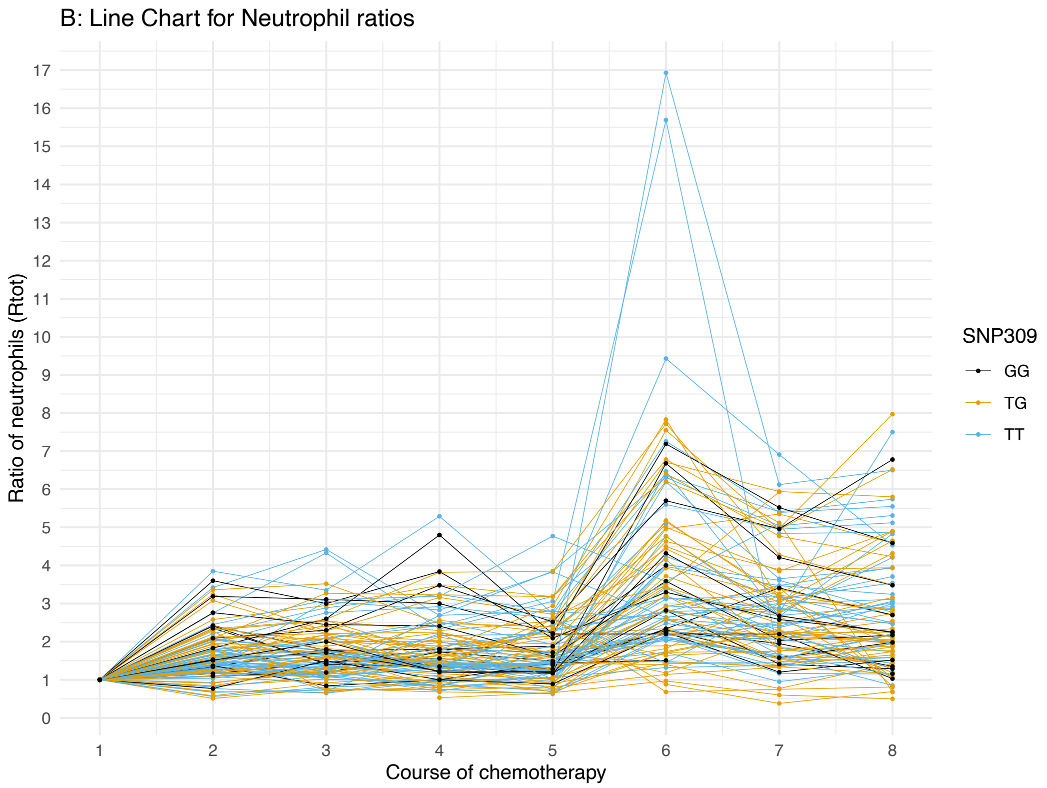

Supplementary Table 7: Impact of *MDM2* SNP309, *MDM2* SNP285 and *MDM2* del1518 status on hematological parameters during chemotherapy. R_{epi} and R_{doc} represent the ratio of cell counts pre and post treatment with epirubicin and docetaxel respectively, while R_{tot} represents the ratio of cell counts before and after completing all courses of chemotherapy.

Groups (test)	Ratios	Leukocytes (p)	Thrombocytes (p)	Hemoglobin (p)
**SNP309**				
TT vs. TG vs. GG	R_{epi}	0.823	0.251	0.794
(Jonckheere-Terpstra)	R_{doc}	0.283	0.970	0.230
	R_{tot}	**0.033**	0.833	0.475
TT vs. TG+GG	R_{epi}	0.997	0.471	0.919
(Mann-Whitney)	R_{doc}	0.288	0.573	0.401
	R_{tot}	**0.041**	0.396	0.893
TT+TG vs. GG	R_{epi}	0.667	0.160	0.352
(Mann-Whitney)	R_{doc}	0.560	0.296	0.212
	R_{tot}	0.180	**0.023**	0.106
**SNP285**				
GC vs. GG	R_{epi}	0.260	0.858	0.881
(Mann-Whitney)	R_{doc}	0.666	0.072	0.914
	R_{tot}	0.413	0.296	0.728
**del1518**				
ii vs. id vs. dd	R_{epi}	0.324	0.744	0.425
(Jonckheere-Terpstra)	R_{doc}	0.505	0.926	0.077
	R_{tot}	**0.043**	0.932	0.165
ii vs. id+dd	R_{epi}	0.210	0.329	0.195
(Mann-Whitney)	R_{doc}	0.592	0.663	0.379
	R_{tot}	0.151	0.480	0.222
ii+id vs. dd	R_{epi}	0.949	0.396	0.738
(Mann-Whitney)	R_{doc}	0.530	0.440	**0.038**
	R_{tot}	**0.048**	0.339	0.349

ii: homozygous insertion genotype
id: heterozygous genotype
dd: homozygous deletion genotype

Supplementary Fig. 3: Dot plot illustrating ratios of neutrophil counts post-treatment/pre-treatment (R_{tot}) for breast cancer patients undergoing neoadjuvant chemotherapy, stratified by all combinations of *MDM2* SNP309, *MDM2* SNP285 and *MDM2* del1518 genotypes in the present dataset. Annotated by order of *MDM2* del1518 (d/d = homozygous deletion genotype, i/d = heterozygous genotype, i/i = homozygous insertion type), *MDM2* SNP285 (GG, GC, no patients with the CC genotype in the present dataset), *MDM2* SNP309 (TT, TG, GG). Grey bars indicate mean value in each group.

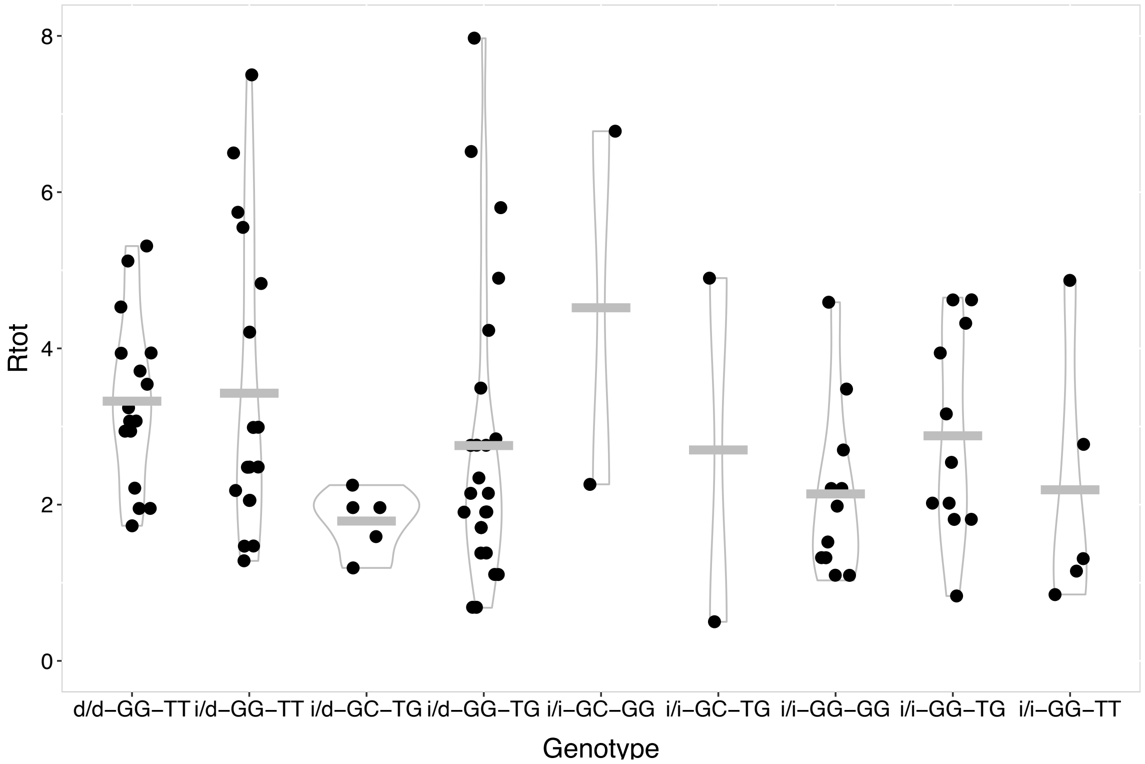

Supplementary Table 8: Combinatorial genotypes of *MDM2* polymorphisms and impact on hematological parameters during chemotherapy. R_{epi} and R_{doc} represent the ratio of cell counts pre and post treatment with epirubicin and docetaxel respectively, while R_{tot} represents the ratio of cell counts before and after completing all courses of chemotherapy.

Groups (test)	Ratios	Leukocytes (p)	Thrombocytes (p)	Hemoglobin (p)
SNP309TT-del1518dd vs. Rest	R_{epi}	0.949	0.396	0.738
(Mann-Whitney)	R_{doc}	0.530	0.440	**0.038**
	R_{tot}	**0.048**	0.339	0.349
SNP309TT-del1518id vs. Rest	R_{epi}	0.378	0.124	0.808
(Mann-Whitney)	R_{doc}	0.385	0.799	0.266
	R_{tot}	0.173	0.647	0.289
SNP309TT-del1518dd+id vs. Rest	R_{epi}	0.430	0.515	0.955
(Mann-Whitney)	R_{doc}	0.232	0.412	0.444
	R_{tot}	**0.008**	0.265	0.906

ii: homozygous insertion genotype
id: heterozygous genotype
dd: homozygous deletion genotype

Supplementary Table 9: Impact of *MDM2* SNP309 status on hematological parameters during chemotherapy, restricted to patients with the SNP285GG genotype (SNP285GC genotype removed to account for potential effect of linkage disequilibrium). R_{epi} and R_{doc} represent the ratio of cell counts pre and post treatment with epirubicin and docetaxel respectively, while R_{tot} represents the ratio of cell counts before and after completing all courses of chemotherapy.

**SNP309 Groups (test)**	**Ratios**	**SNP285GC removed**			
		**Neutrophils (p)**	**Leukocytes (p)**	**Thrombocytes (p)**	**Hemoglobin (p)**
TT vs. TG vs. GG (Jonckheere-Terpstra)	R_{epi}	0.917	0.876	0.282	0.893
	R_{doc}	0.201	0.292	0.483	0.307
	R_{tot}	**0.026**	**0.029**	0.727	0.706
TT vs. TG+GG (Mann-Whitney)	R_{epi}	0.763	0.749	0.495	0.923
	R_{doc}	0.25	0.351	0.288	0.381
	R_{tot}	**0.046**	0.062	0.241	0.949
TT+TG vs. GG (Mann-Whitney)	R_{epi}	0.386	0.301	0.161	0.480
	R_{doc}	0.386	0.426	0.656	0.481
	R_{tot}	0.084	0.052	0.086	0.324

Supplementary Table 10: Impact of *MDM2* SNP285 status on hematological parameters during chemotherapy, restricted to patients with the SNP309GG or TG genotype (SNP309TT genotype removed to account for potential effect of linkage disequilibrium). R_{epi} and R_{doc} represent the ratio of cell counts pre and post treatment with epirubicin and docetaxel respectively, while R_{tot} represents the ratio of cell counts before and after completing all courses of chemotherapy.

**SNP285 Groups (test)**	**Ratios**	**SNP309TT removed**			
		**Neutrophils (p)**	**Leukocytes (p)**	**Thrombocytes (p)**	**Hemoglobin (p)**
GC vs. GG (Mann-Whitney)	R_{epi}	0.286	0.259	0.959	0.825
	R_{doc}	0.967	0.983	**0.031**	0.958
	R_{tot}	0.802	0.820	0.187	0.702

Supplementary Table 11:
Comparisons of neutrophil counts between SNP309 genotypes, pre treatment and post treatment with chemotherapy.

**Groups (test)**	**Neutrophils (pre treatment) (p)**	**Neutrophils (post treatment) (p)**
**SNP309**		
TT vs. TG vs. GG (Jonckheere-Terpstra)	0.871	0.232
TT vs. TG+GG	0.773	0.542

(Mann-Whitney)		
TT+TG vs. GG (Mann-Whitney)	0.608	0.088
**Combinatorial genotypes**		
SNP309TT-del1518dd vs. Rest (Mann-Whitney)	0.974	0.123
SNP309TT-del1518id vs. Rest (Mann-Whitney)	0.301	0.245
SNP309TT-del1518dd+id vs. Rest (Mann-Whitney)	0.405	**0.031**
